# The effect of compression and combined compression-tactile stimulation on lower limb somatosensory acuity

**DOI:** 10.3389/fspor.2023.1235611

**Published:** 2023-10-17

**Authors:** Ashleigh Marchant, Sarah B. Wallwork, Nick Ball, Jeremy Witchalls, Gordon Waddington

**Affiliations:** ^1^ Research Institute for Sport and Exercise, University of Canberra, Canberra, ACT, Australia; ^2^ IIMPACT in Health, University of South Australia, Adelaide, SA, Australia

**Keywords:** compression garment, cutaneous feedback, proprioception, somatosensation, tactile sensation

## Abstract

**Background:**

Lower limb somatosensation and proprioception are important for maintaining balance. Research has shown that compression garments or exposure to textured surfaces, can enhance somatosensation however, little is known about the effect of combined compression and texture on somatosensory acuity in the lower limb. This study aimed to assess the effects of combined compression socks with a plantar textured sole, on lower limb somatosensory acuity.

**Methods:**

Thirty participants completed a somatosensory acuity task (active movement extent discrimination apparatus; AMEDA) under three conditions: barefoot (control condition), standard knee-high compression sock (compression sock), and knee-high compression sock with internal rubber nodules situated on the sole (textured-compression sock). Somatosensory acuity was assessed between the different sock conditions for the (i) entire group, (ii) high performers, and (iii) low performers. It was hypothesized that low performers would see gains wearing either sock, but the greatest improvement would be in the textured-compression sock condition.

**Results:**

AMEDA scores were not significantly different between conditions when the entire group was analyzed (*p *= 0.078). The low performers showed an improvement in somatosensory acuity when wearing the compression sock (*p *= 0.037) and the textured compression sock (*p* = 0.024), when compared to barefoot, but there was no difference between the two sock conditions (*p* > 0.05). The high performers did not show any improvement (*p* > 0.05 for all).

**Conclusion:**

These findings demonstrate that additional sensory feedback may be beneficial to individuals with lower baseline somatosensory acuity but is unlikely to provide benefit for those with higher somatosensory acuity.

## 1. Introduction

Somatosensation is a collective term in which tactile and proprioceptive information are used to establish the position of our limbs in space (1). Somatosensory acuity refers to the precision to which one can correctly identify body position and location. It contributes essential feedback to the mechanisms that maintain balance in an upright and purposeful, stable position. Proprioceptive acuity, or proprioception, is described as the ability to accurately identify joint position, where our limbs gain a sense of positioning through acting velocities and forces, including gravity (1). Activation of muscle spindles and golgi tendon organs alert the central nervous system if a muscle or tendon is stretched, thereby identifying the position of the joint relative to the rest of the body (1, 2). Specifically, ankle proprioception is important for postural balance, gait pattern and overall ability to maintain an upright posture. Poor ankle proprioception may influence the rest of the kinetic chain and is associated with increased vulnerability to lower limb injury (3–5).

While good ankle somatosensory acuity may reduce the risk of ankle injury, in turn, injury can also be associated with poor somatosensory acuity (3, 6). For example, individuals with chronic ankle instability present with reduced performance on an active ankle proprioception assessment, and individuals with an anterior cruciate ligament (ACL) injury have less precision on a joint position sense test than those without injury (7, 8). A large portion of the current literature has researched interventions to address proprioceptive deficits following injury, due to a number of medical conditions or increasing age. Interventions to increase proprioceptive acuity and somatosensation in a healthy, or unrestricted population may also be useful in athletes, military personnel, astronauts, or even individuals looking to reduce their risk of injury. Exposure to exercises which promote balance and careful neuromuscular control have been shown to improve somatosensory acuity (9, 10) and in particular, wobble board training has been demonstrated as effective in improving ankle somatosensory performance among dancers, the elderly, Australian rules footballers, and Rugby League players (11–14). However, while physical exercise is shown to enhance somatosensation, several weeks are required for the somatosensory system to adapt to the training. In some populations, there is a necessity for a more acute stimuli for somatosensory adaptations to promote immediate, continued, or enhanced function. For example, post-surgery, extensive bed rest, return to sport, older adults at risk of falling, or mobilizing on uneven terrain, are just some instances where a rapid strategy could be useful (15, 16). One acute intervention commonly used is a textured insole and has been shown to provide immediate improvements in lower limb somatosensory acuity, decrease postural sway, and improve postural balance (17–21). Compression garments have also been shown to have a positive impact on somatosensory acuity and increase one's ability to detect small changes in joint movement (22, 23). It has been suggested that additional sensory input, can modulate the signal to the central nervous system, thereby providing more filtered feedback to accurately identify limb position (24). This additional sensory input introduces variability that acts as a functional regulator, encouraging exploratory behavior to enhance perception and refine motor control (25). This phenomenon appears to hold true even in healthy and unrestricted individuals (14, 21, 22) It is not known whether further stimulation in the form of combined lower limb compression and textured insoles may augment lower limb somatosensory acuity.

While it is clear that additional tactile feedback to the foot and ankle (compression garment or textured insoles) is advantageous to joint position sense, recent studies have shown that it may only benefit those with poor lower limb somatosensory acuity (19, 22, 26). That is, individuals who perform below average on a task that measures foot and ankle somatosensory acuity (active movement extent discrimination apparatus; AMEDA) see improvements in somatosensory acuity when provided with additional tactile feedback, but those with above average scores do not. The AMEDA measures somatosensory acuity by assessing an individual's ability to discriminate between varying degrees of joint position (27). It is a well-established assessment tool within ankle somatosensation and provides a numerical value of performance. Ankle instability has also proven to be a factor in ankle somatosensory acuity, whereby those who display chronic weakness or recurring pain in the ankle joint perform poorly on the AMEDA (19, 28). However, it is uncertain whether a combination of tactile insole and compression garments would affect this population of lower performers.

**Figure 1:**
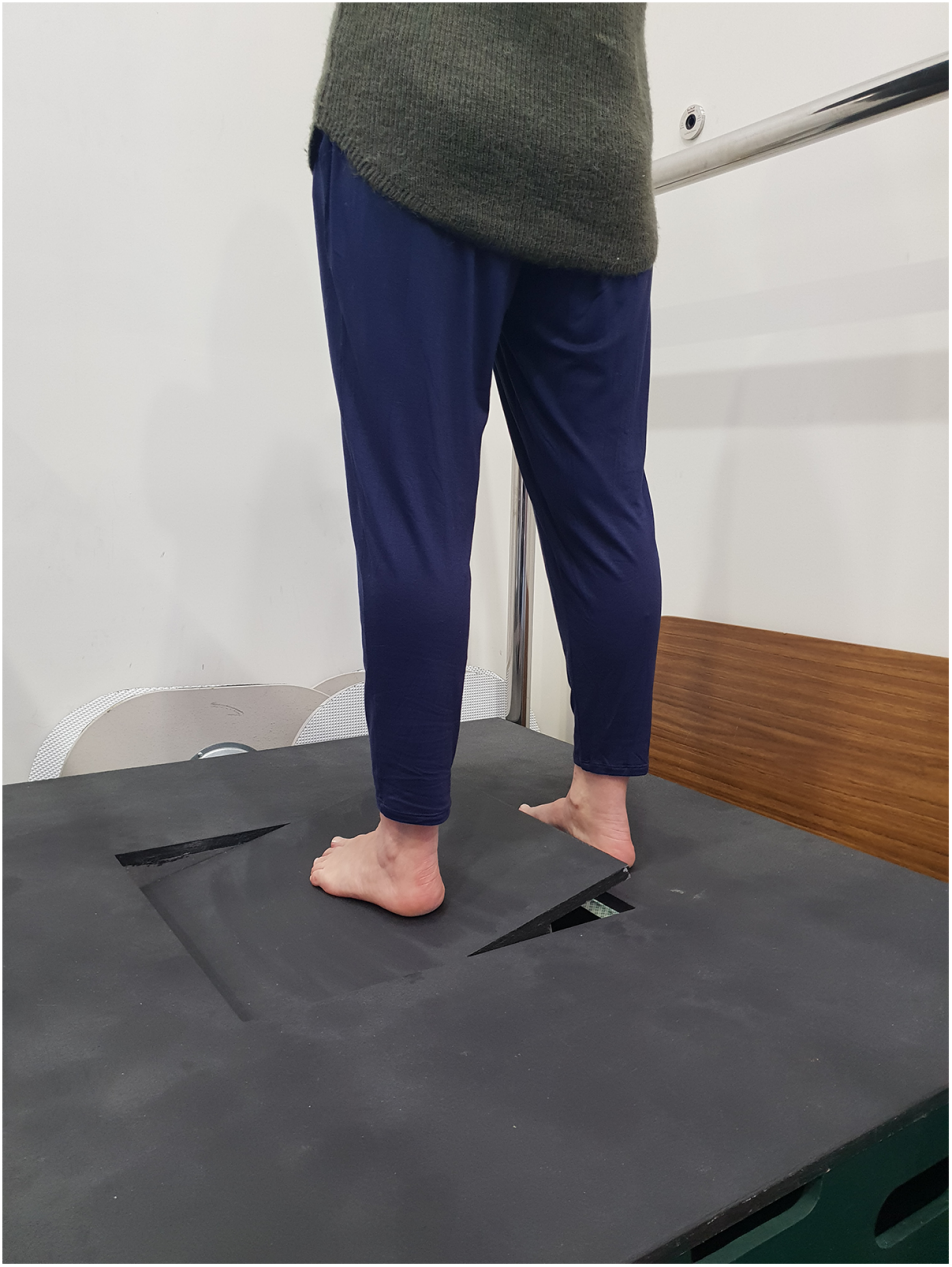
The active movement extent discrimination apparatus (AMEDA) assesses the ability to judge ankle inversion and eversion movement.

The primary aim of this study was to explore the effect of combined lower limb compression and plantar tactile stimulation on ankle somatosensation in unrestricted adults. Lower limb somatosensory acuity was measured under three conditions using the ankle AMEDA (Figure 1): (i) no sock (barefoot), (ii) standard knee-high compression sock (compression sock), and (iii) knee-high compression sock with a textured inner sole lining (textured-compression sock). We hypothesized that AMEDA scores would be greater (indicating better somatosensory acuity) in the sock conditions compared to no sock condition, and the textured-compression sock would provide the greatest acuity score of the three. To gain a more detailed assessment of this group, our secondary aim was to explore the effect of the additional tactile stimulation (compression and plantar tactile stimulation) in those with baseline above average somatosensory acuity and those with baseline below average somatosensory acuity within this study population. We hypothesized that low performers (participants with below average barefoot) scores would see gains in AMEDA scores in both sock conditions, however these gains were unlikely to be observed in high performers (participants with above average baseline scores).

## 2. Materials and methods

### 2.1. Participants

Thirty-two unrestricted participants between the ages of 18 and 65 years were recruited for this study. To achieve a statistical power of >0.80, data analytic software G*Power (RRID:SCR_013726) was used to determine that we required at least thirty-one participants. This was based on a medium effect size (*d* = 0.46) guided by previous research which assessed repeated AMEDA scores across various interventions among young adults (8, 28, 29). The study was completed in accordance with the University of Canberra Human Research Ethics Statement (reference number: 20210236). To be considered unrestricted, participants had to present with free movement at the ankle, without any restrictions that impacted day to day tasks. Exclusion criteria were any medical condition which may affect balance, or any ankle injury within the previous 3 months. Participants attended the laboratory for a single 40-minute session. Written informed consent was obtained prior to participation.

### 2.2. Sock conditions

**Figure 2:**
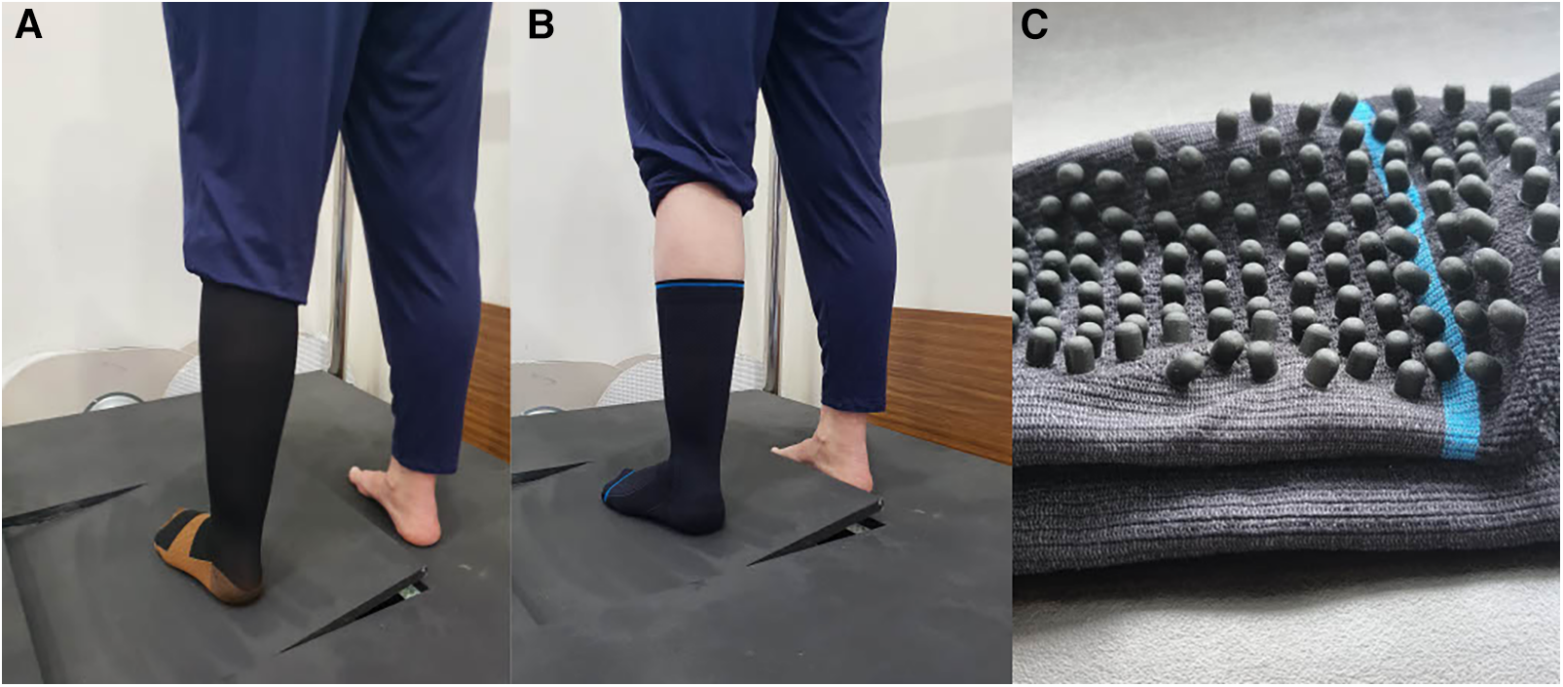
Standard compression sock (**A**), textured-compression sock (**B**), image highlighting the rubber nodules within the textured-compression sock (**C**): when worn, the nodules are on the inner surface of the sock and in contact with the plantar aspect of the foot (i.e., touching the skin).

Somatosensory acuity was measured across three conditions: (i) barefoot, (ii) standard compression sock (compression sock), and (iii) combined compression sock with a textured inner sole (textured-compression sock). The barefoot condition was used as a control condition. The compression socks were purchased online from LAFUYSO (Retrieved from https://www.amazon.com.au/stores/LAFUYSO) and had a compression rating of 20–30 mmHg. When worn, the sock reached to approximately the head of fibula. The textured-compression sock was provided by SRCHealth Pty Ltd for the purpose of this study and had a compression rate of 20–30 mmHg. These reached to approximately 50 mm below the head of the fibula. In addition to the compression, rubber nodules were fixed to the inside of the sock and were in direct contact with the plantar surface of the foot (i.e., touching the skin). See Figure 2, for an image of both sock conditions. The order in which participants wore the socks was randomized using an online number generator (https://www.random.org/).

### 2.3. Outcome measures

#### 2.3.1. Active movement extent discrimination apparatus (AMEDA)

The AMEDA task requires participants to make an absolute judgement about joint position by utilizing proprioceptive feedback in a functional manner (30). The ankle AMEDA (Figure 1) was used to assess participant somatosensory acuity of the foot and ankle (27, 30). Participants were asked to stand with one foot (non-testing side) on a solid platform and the other (testing side) on a moving platform and rotate the moving platform to one of five pre-defined depths (varying degrees of ankle inversion). The order in which the five depths were presented was pseudorandomized. Participants generated and controlled the velocity of the inversion movement and were asked to rotate the platform until they felt it stop and then return to neutral (horizontal). They were asked to state which position they had experienced (i.e., position 1 through to position 5, with 1 being the smallest depth of inversion) and were asked to maintain their gaze straight ahead to avoid visual feedback regarding ankle position. Each depth of ankle inversion had 1° of difference between positions (position 1: 10.5° from horizontal, position 5: 14.5° from horizontal). Participants undertook several of these judgements to then produce a numerical score on how well they can discriminate small changes of joint differences; thereby providing an evaluation of their proprioceptive acuity (27, 30). Participant responses for each AMEDA assessment were uploaded to a Microsoft Excel spreadsheet (Microsoft Corporation. 2018. Microsoft Excel. Retrieved from https://office.microsoft.com/excel). A matrix of correctly identified positions was then created to produce an Area Under the response Curve (AUC) score. A score between 0.5 (equivalent to chance) and 1.0 (equivalent to a perfect score) was generated for each participant and each condition to signify the participant's sensitivity to the test. Participants therefore had three scores, representative of each condition (barefoot, compression sock, textured-compression sock). A low AMEDA score has been associated with conditions which impact proprioception, such as ankle injury (4, 28), while a high score is associated with high performance such as elite sports (4, 31).

#### 2.3.2. Cumberland instability questionnaire (CAIT)

The Cumberland Instability (CAIT) questionnaire was used to establish presence of ankle instability as a confounding factor. While participants were considered unrestricted, CAI may have still been present and may impact lower limb somatosensory acuity (19, 28). The CAIT is a reliable nine-item graded questionnaire that prompts users to rate their level of pain and instability during different tasks including walking, running, and jumping. Each question has between 3 and 5 possible answers and participants receive a score out of 30 for each ankle, with a lower score indicating a higher degree of ankle instability. Hiller et al. (32) identified a score of 27 (out of a possible 30) or below as indicating that the participant likely has ankle instability. More recent data however has revised that a score of 25 or below is a more accurate indicator of able instability (33). Using a cut off score of 25, Wright et al. (33) demonstrated a sensitivity and specificity percentage of 96.6 and 86.8, respectively. The revised score of 25 and below was therefore used in the present study.

### 2.4. Procedure

After written informed consent was signed and the CAIT completed, participants undertook three assessments on the ankle AMEDA. Participants were first asked which their preferred kicking foot was. and testing was completed on the opposite side as the stabilizing leg, also known as the stance leg, has been shown to have higher proprioception than the kicking leg (34). Participants were required to wear each of the socks across the three AMEDA assessments (barefoot, compression sock, textured-compression sock).

Familiarization with the AMEDA was completed prior to the first ankle AMEDA assessment only. Familiarization involved exposure to the five levels of inversion in a sequential order, three times. The formal assessment commenced after the familiarization period. Participants were asked to verbalize which position they had experienced (i.e., position 1, 2, 3, 4 or 5). Each AMEDA assessment included 50 inversion ankle movements, or ’stops’, for a total of 150 across the three tests. ’Stops’ were pseudorandomized so that each position was presented 10 times during one assessment, but in a simulated random order. Each AMEDA assessment took approximately 6–7 min to complete. Between AMEDA assessments, all participants were asked to remove the sock (if wearing one) and walk across the room and back (approximately 20 meters) before commencing the next assessment. This was introduced to ‘reset’ sensory changes between assessments. A more prolonged break was offered in between conditions at the participants’ discretion.

### 2.5. Statistical analysis

SPSS statistics (IBM Corp. Released 2020. IBM SPSS Statistics for Windows, Version 27.0. Armonk, NY: IBM Corp) was used to analyze AMEDA AUC scores with an alpha 0.05 used to determine statistically significant results. The data was tested for normality via a Shapiro–Wilk test. A one-way repeated measures analysis of variance (ANOVA) was conducted to assess whether there was any change among the sequence of AMEDA tests that could signify a learning effect. An independent t test was conducted to determine whether there was any difference between those with and without chronic ankle instability (CAI) as established by the CAIT questionnaire.

#### 2.5.1. AMEDA analysis: entire group

To address our primary aim of comparing AMEDA scores across the three conditions and within the entire group, a one-way repeated measures ANOVA was conducted (3 levels: barefoot, compression sock, textured compression sock). Post-hoc paired t-tests were conducted to further explore any significant effects.

#### 2.5.2. AMEDA analysis: high and low performers

To address our secondary aim of comparing AMEDA scores across the three conditions in the high and low performers, participants were first spilt into the two groups according to their baseline performance. Baseline performance was considered as the AMEDA AUC score achieved whilst barefoot. High performers were those with a baseline AMEDA AUC score greater than the mean score of this population group. Low performers were those with a baseline AMEDA AUC score less than the mean score of this population group. We chose to analyze results within the current study population, rather than across the broader population. While this may reduce the precision in defining good or poor performers, it provides valuable insight into how the current study group responded to the stimuli. Similar techniques have been used in prior studies, where the division was based on the population rather than a specific value, and we have adopted a similar approach (22). A 3 × 2 repeated measures ANOVA was then conducted to compare scores on the AMEDA between conditions (3 levels: barefoot, compression sock, and textured compression sock) and performance group (2 levels: low performers and high performers). Post-hoc paired t-tests were conducted to further explore any significant effects.

## 3. Results

### 3.1. Participant characteristics

Thirty-two participants were recruited. Two data outputs were corrupt at analysis and as such, our final sample size was 30 which provided a statistical power of 0.79. Demographics of the sample population are presented in Table 1. Nine of the 30 participants recalled a history of at least one ankle sprain (on the testing foot) in their lifetime and 7 were uncertain. The CAIT results indicated 6 were likely to currently have CAI. An independent *t*-test showed there was no difference between baseline (barefoot) ankle somatosensory scores for likely CAI and unlikely CAI participants [*t* (28) = 0.69, *p* = 0.49], however the sample size was small (*n* = 6). A one-way repeated measures ANOVA showed that there was no order effect [Wilks’ Lambda = 0.975, F (2,27) = 0.340, *p* = 0.714, multivariate partial eta squared = 0.0.025]. The data were normally distributed [W(0.982), *p* = 0.872].

**Table 1:** Characteristics of the sample population.

Characteristic	Result
Gender	M 16 F 14
Age (M ± SD)	33 ± 13 years
Height (M ± SD)	175 cm ± 11
Weight (M ± SD)	72 kg ± 13
Preferred kicking foot	Right: 27 Left: 3
History of ankle sprain in testing foot	9 (+7 uncertain)
CAIT questionnaire (M ± SD)	27 ± 4.5
Likely to have CAI	6

CAI, chronic ankle instability; M, mean; SD, standard deviation.

### 3.2. AMEDA performance between conditions: entire group

The mean and standard deviation (SD) of AMEDA AUC scores for the entire group were as follows; barefoot: (AUC, SD) 0.68 ± 0.06, compression sock: 0.69 ± 0.05, textured-compression sock: 0.70 ± 0.05. There was no significant main effect of “condition” on AMEDA AUC scores when the entire group was analyzed [Wilks’ Lambda = 0.828, F (2,27) = 2.811, *p* = 0.078, multivariate partial eta squared = 0.172].

### 3.3. AMEDA performance between conditions: high and low performers

**Figure 3:**
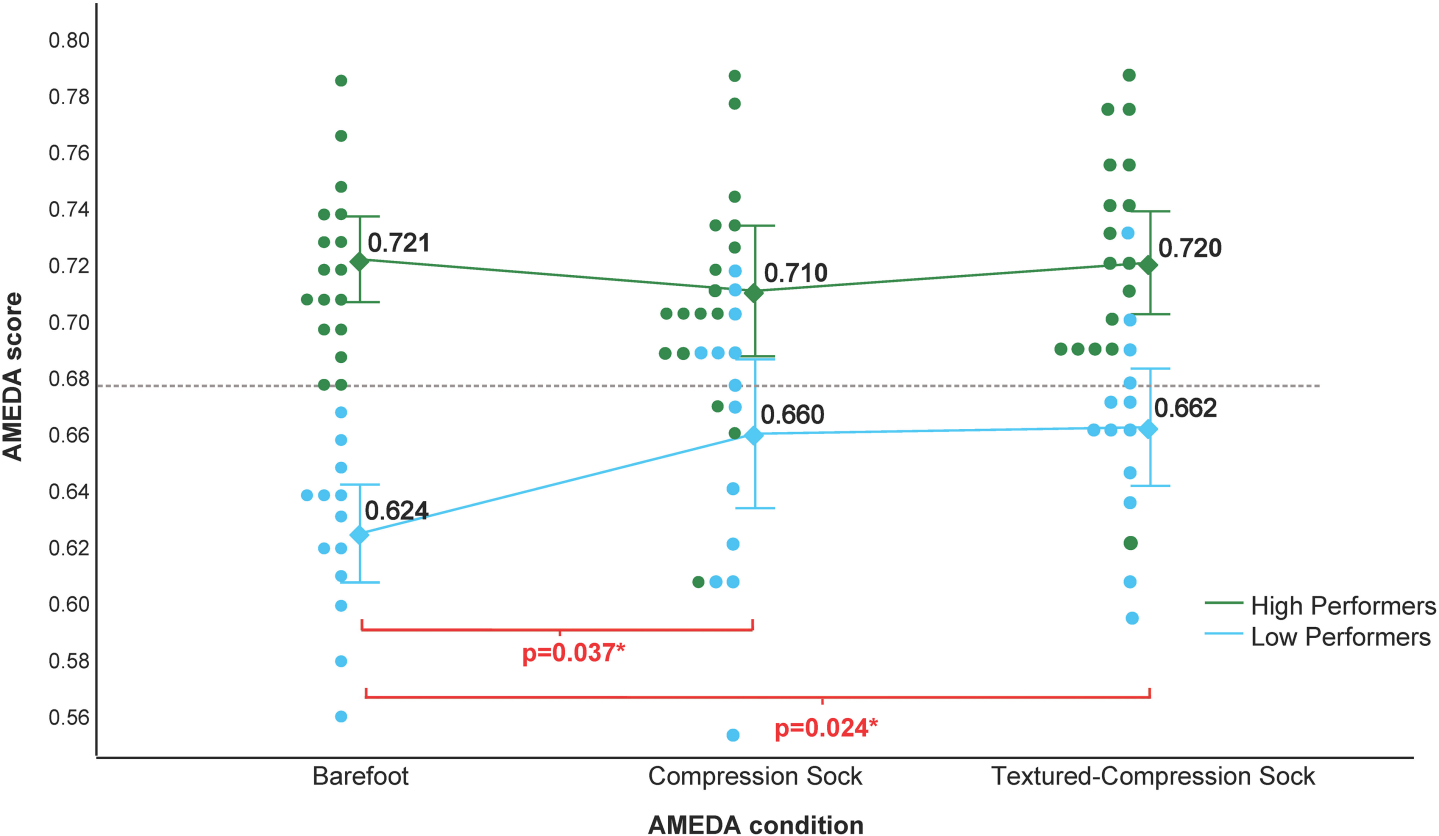
Results of the ankle AMEDA when grouped as high (green) and low (blue) performers. Responses are represented as an AUC score between 0.5 (chance) and 1.0 (perfect score). Barefoot AMEDA scores of the entire cohort (illustrated by the individual points on the left scatter plot) had a mean score of 0.68 and was used to group the study population. A barefoot score over 0.68 was considered high performance and a barefoot score under 0.68 was considered low performance. Error bars represent 95% Confidence Interval. There was no significant difference between conditions among high performers. There was a significant difference among low performers for the compression sock and the textured-compression sock when compared to barefoot, but no difference between socks.

Using the mean barefoot baseline AMEDA AUC score of 0.68, participants were grouped into low performers (baseline score below 0.68, *n* = 13) and high performers (baseline score of 0.68 and above, *n* = 17). Figure 3 shows the mean AMEDA scores of both groups. There was a significant main effect of ‘group’ (high performers vs. low performers) [F (1, 28) = 55.823, *p* < 0.000, multivariate partial eta squared = 0.666], and a significant main effect of ‘condition’ (barefoot vs. compression sock vs. textured-compression sock) [F (2,27) = 4.898, *p* = 0.015, multivariate partial eta squared = 0.266].

Post-hoc t-tests revealed that, in the low performer group, there was a significant difference between the barefoot vs. compression sock condition [*t* (12) = 2.345, *p* = 0.037] and the barefoot vs. textured-compression sock condition [*t* (12) = 2.575, *p* = 0.024]. There was no significant difference between the compression and textured compression sock (*p* = 0.901). In the high performer group, there were no differences between any conditions (barefoot vs. compression sock *p* = 0.316; barefoot vs. textured-compression sock *p* = 0.894; compression sock vs. textured-compression sock *p* = 0.438).

## 4. Discussion

This study aimed to explore the effect of combined lower limb compression and plantar tactile stimulation on ankle somatosensation in unrestricted adults. We hypothesized that performance would be greatest (i.e., a higher score on the ankle AMEDA) in the condition of textured-compression sock. We found that when all participants were analyzed as an entire group, there was no significant difference between the three conditions. However, when participants were grouped according to their baseline AMEDA scores, those classified as low performers, improved in both sock conditions when compared to baseline. This enhanced performance was not seen in the higher performers.

Until now, there has been limited research available on how the combination of compression with a tactile stimulus may affect lower limb somatosensation. Instead, previous studies have explored the effects of a single intervention and demonstrated that a compression garment alone (22, 23, 35), or textured insoles alone (17–21) can improve active joint repositioning tasks in the lower and upper limb. Authors have theorized that an increase in sensory stimulation boosts the ability to recruit afferent axons and offers greater feedback to the nervous system, enabling a more accurate judgement on joint position (24, 35). Additionally, the distribution of receptors in the plantar aspect of the foot are crucial to maintain one's center of mass (36, 37) and researchers speculate that supplemental sensory feedback aids in filtering receptor information (24). Therefore, we theorized that with even greater stimulation (i.e., the combined texture and compression) the feedback loop would further enhance somatosensory acuity. However, we found that this additional stimulation did not further enhance somatosensory acuity, as measured on the AMEDA. A previous upper limb AMEDA study has suggested that finger somatosensory acuity in healthy adults may be sensitive to overloading and, in some instances, too much incoming signal may cause overstimulation and reduced joint position sense (38). Further, research on patients with patellofemoral pain syndrome demonstrated that somatosensory acuity of the knee is greater when the person is in a non-weightbearing posture compared to standing upright, in a “normal” weight bearing status (39). It is hypothesized that when upright, there is too much signal “noise” in people with pain and the central nervous system struggles to differentiate between various knee positions. In our study, somatosensory acuity did not reduce but instead remained unchanged which perhaps suggests that there is a point where too much afference signal is not helpful to detect changes in joint position.

In this study, we found that the wearing a sock improved somatosensory acuity in low performers. The phenomenon is also observed in a study by Steinberg et al. (26), where ballet dancers completed the ankle AMEDA under four conditions of varied footwear (barefoot, ballet shoe with no insole, ballet shoe with smooth insole, ballet shoe with textured insole). The participants were grouped into tertiles based on their score obtained whilst wearing their ballet shoes. They found that the additional feedback textured insole improved somatosensory acuity on the AMEDA, but only for those who had lower baseline score. A low AMEDA score can be associated with not only ankle instability but slowed rate of learning during a repeat ankle AMEDA test (28). So, ability to increase ankle somatosensory acuity through the simple task of wearing a specialized sock may be useful to those at risk of injury, falls, or mobilizing on novel terrain. In our study, poor baseline scorers (when ranked as being in the bottom 50% of barefoot scores) significantly improved their AMEDA scores whilst wearing either type of sock, with some individual outliers matching or even surpassing those in the high performer group. However, there was no significant difference between the two sock conditions, even within the low performers, confirming that additional stimulation does not necessarily further enhance somatosensory acuity.

In the present study, high performers did not demonstrate any improvement in somatosensory acuity in either sock condition. High somatosensory acuity has been associated with elite athletes as the person has a good sense of body awareness (40, 41). Muaidi et al. (41) suggest the superior ability may be an innate sensory skill which led these people to excel in the sport through natural selection, or it could be attributed to years of training in such a repetitive environment. Either way, it implies that somatosensation can be an internalized task, and in higher performers an additional, external sensory resource, (i.e., a sock), is not useful. Research into performance augmentation involving compression garments has demonstrated that additional feedback may in fact inhibit sensitivity for an individual who has better than average baseline somatosensation (22). While results of the current study were not reduced but instead unchanged, it does demonstrate that additional feedback is of no use for somatosensory acuity for some individuals. Broatch et al. (22) theorize that in such a situation, there is too much “noise” for somebody who presents with high body awareness. It may be that somatosensation has a ceiling effect or point where additional feedback does not provide any further enhancement to performance (42). We saw evidence of this in the current study where high performers received no benefit to wearing either sock compared to barefoot, but also in the low performers where there was no further enhancement in the textured-compression sock. We found no overall group effect of ’sock condition’, perhaps because any effects seen in the low performers were ‘washed out’ by the lack of improvement seen in the high performers.

When comparing results of the CAIT questionnaire to AMEDA score, there was no significant difference between those with and without ankle instability. Instead, the gains from wearing either sock were only apparent for those who under performed on the AMEDA at baseline with no regard to ankle instability status. Interventions such as tactile stimulating accessories and garments, or exercise therapy to improve somatosensation is not a new concept and often used to reduce the risk of injury, particularly for those who suffer from ankle instability. In a project by Steinberg et al. (11) researchers added a textured surface to the traditional balance board training, historically completed with a smooth surface. The authors found that dancers with a history of ankle injury were able to gain from the training at a faster rate than those without. However, it is important to note that our sample size of individuals with ankle instability as determined by the CAIT was small (*n* = 6) as this was not the main aim of this project. Interestingly, the ankle instability population were split evenly across the high and low performers. Future investigations could be aimed at determining whether training regimes are perhaps more relevant to be directed to those who have poor somatosensory instead of basing it on ankle stability status.

### 4.1. Limitations

A limitation of this research is that we were not adequately powered for our secondary analyses and therefore these findings need to be interpreted with caution. That is, the division of participants into high and low performers was unequal and had small sample sizes (13 low performers, 17 high performers), meaning that our analyses were underpowered. Future research specifically investigating the influence of compression and tactile socks in ‘poor performers’ is important to confirm the findings of this study. Furthermore, the order of testing was pseudo-randomized to ensure the order of the conditions was equal across the entire sample. This was not carried across to the high and low performance groups and therefore we cannot be certain that the order of conditions has not had an effect. Additionally, the purpose of this study was to assess whether the combination would further enhance AMEDA scores compared to a plain compression sock. However, without a texture-only matched condition it is not possible to determine whether the maintenance of performance in the combined condition is due to compression-only, without additional gains from the texture, or whether it represents a ceiling effect where a weighted combination of compression and texture reaches a peak of enhanced performance. Future studies could use a plain sock with planter rubber nodules alongside a plain compression sock for an additional comparison. Further, as the socks were only worn for the duration of the assessment, it is unclear what effect they have on performance outside of this, or longer-term. Future research should investigate whether wearing the compression sock with textured in-sole for longer durations influences somatosensory acuity.

## 5. Conclusion

Results from this study raise the possibility that a plain compression sock, and a novel sock combining the effects of compression and textured insole lining, may improve short-term ankle somatosensation in unrestricted individuals with low baseline somatosensory acuity, compared to barefoot. This improvement was not observed among individuals with high baseline somatosensory acuity. It suggests that the additional feedback may be a useful tool for low performers and even has potential to increase somatosensory awareness on the lower limb AMEDA in some individuals to levels equivalent of those with naturally high acuity. Notably, there was no significant difference between the plain compression sock and the mixed textured-compression sock. It remains unclear whether a ceiling effect may be occurring and performance plateaus, or if the additional stimulation from the nodules was not useful. Future research should include a plain textured sock for comparison, and involve larger participant groups, particularly in the low performers to validate our findings. Additionally, we suggest when using the AMEDA in future intervention studies, researchers should consider whether it is potentially valuable to categorize participants based on their baseline barefoot acuity score.

## Data Availability

The raw data supporting the conclusions of this article will be made available by the authors, without undue reservation.

## Supplementary material

Supplementary Figure 1 Example of two participant ankle AMEDA results. Participant 4 (p4) was within the low performer group as their barefoot score was 0.56 (<0.68). This individual continued to increase their somatosensory acuity on the AMEDA with each level of tactile stimulation. Participant 15 (p15) was within the high performer group as their baseline score was 0.73 (>0.68). Their score was unchanged from barefoot to textured-compression sock and reduced by 0.03 points whilst wearing the compression sock. Both individuals completed the same test sequence.
