# Supplementary material for: The effect of compression and combined compression-tactile stimulation on lower limb somatosensory acuity
Source: Front Sports Act Living. 2023 Oct 17;5:1235611. doi: 10.3389/fspor.2023.1235611 (PMC10622748; doi:10.3389/fspor.2023.1235611)
Supplement: Supplementary Figure 1 — Example of two participant ankle AMEDA results. Participant 4 (p4) was within the low performer group as their barefoot score was 0.56 (<0.68). This individual continued to increase their somatosensory acuity on the AMEDA with each level of tactile stimulation. Participant 15 (p15) was within the high performer group as their baseline score was 0.73 (>0.68). Their score was unchanged from barefoot to textured-compression sock and reduced by 0.03 points whilst wearing the compression sock. Both individuals completed the same test sequence. [file Image1.pdf]

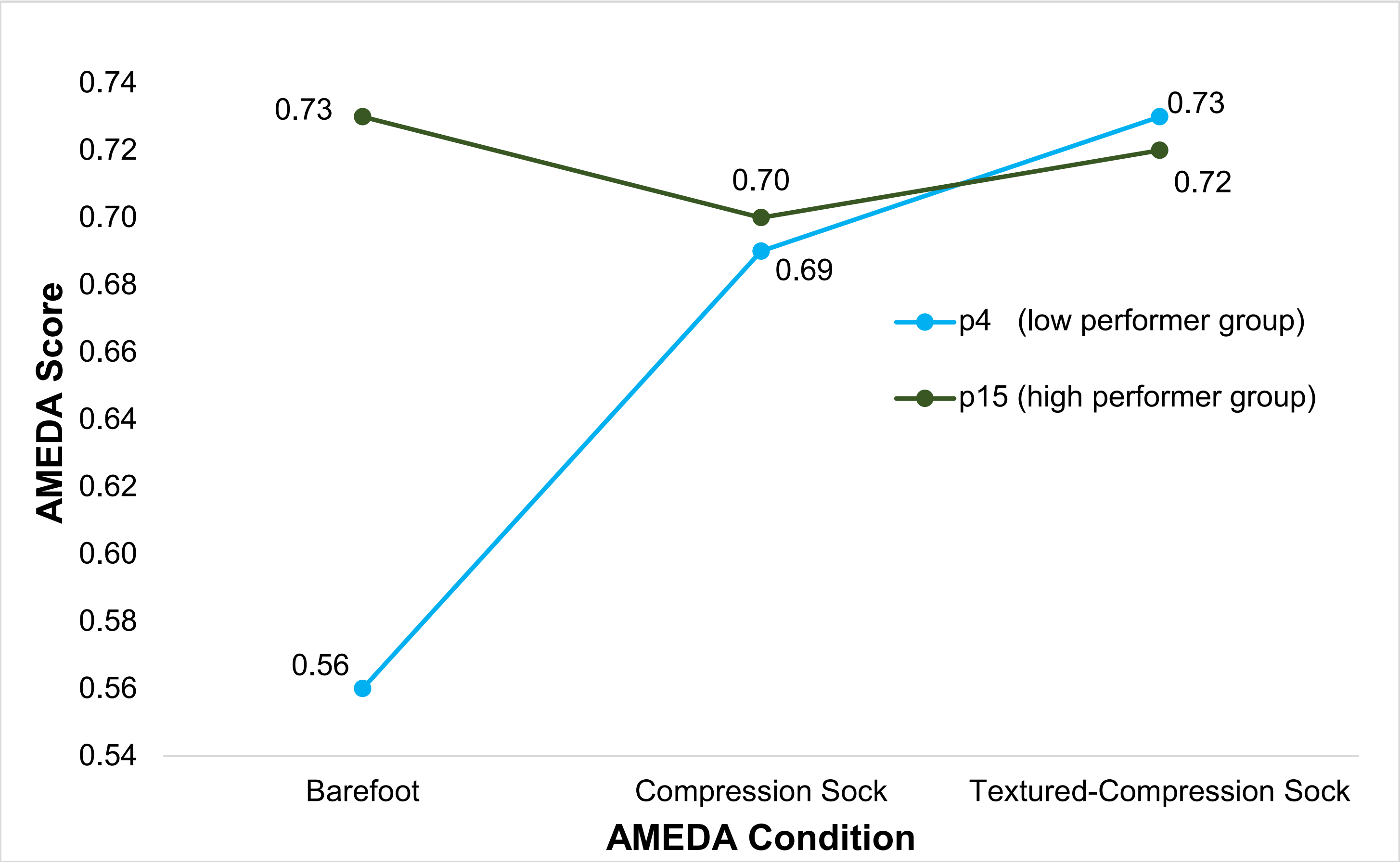

Figure 4: Example of two participant ankle AMEDA results. Participant 4 (p4) was within the low performer group as their barefoot score was 0.56 (<0.68). This individual continued to increase their somatosensory acuity on the AMEDA with each level of tactile stimulation. Participant 15 (p15) was within the high performer group as their baseline score was 0.73 (>0.68). Their score was unchanged from barefoot to textured-compression sock and reduced by 0.03 points whilst wearing the compression sock. Both individuals completed the same test sequence.
